# Identification and whole-genome characterization of a novel equine papillomavirus

**DOI:** 10.1007/s11262-025-02190-y

**Published:** 2025-10-23

**Authors:** Anne-Lie Blomström, Sanni Hansen, Miia Riihimäki

**Affiliations:** 1 Department of Animal Biosciences, Swedish University of Agricultural Sciences, Uppsala, Sweden; 2 Department of Veterinary Clinical Sciences, Faculty of Health and Medical Sciences, University of Copenhagen, Copenhagen, Denmark; 3 Department of Clinical Sciences, Swedish University of Agricultural Sciences, Uppsala, Sweden

**Keywords:** Equine papillomavirus, Genetic characterization, Complete genome

## Abstract

Papillomaviruses (PVs) are small, non-enveloped viruses with double-stranded circular DNA genomes that infect a wide range of hosts, including mammals, birds, reptiles, and fish. While human papillomaviruses are extensively studied, recent advancements in high-throughput sequencing techniques have increased the detection and genetic characterization of PVs from various animal species. Here, we describe the identification and whole-genome characterization of a divergent equine papillomavirus (EcPV) detected through a viral metagenomic investigation of a horse in Denmark exhibiting neurological signs. Using Nanopore sequencing and Sanger sequencing, we assembled a complete viral genome of 7767 nucleotides. Phylogenetic analysis, based on concatenated E1, E2, L2, and L1 gene sequences, showed that the identified virus clustered within the same clade as EcPV3 (genus *Dyoiotapapillomavirus*) and EcPV6 (genus *Dyorhopapillomavirus*) but was situated on a distinct separate branch. Comparative genome analysis revealed approximately 52% nucleotide sequence similarity to EcPV3 and EcPV6, which share 66% similarity with each other. The L1 gene, commonly used for papillomavirus classification, exhibited a sequence identity to EcPV3 (58.4%) and EcPV6 (60.0%). The other viral genes displayed a 39–62% identity to the respective genes from EcPV3 and 6 further supporting the divergence of this newly identified PV. The combination of the phylogenetic analysis and the genetic divergence suggests that this newly identified papillomavirus may constitute a novel species or genus within the *Papillomaviridae* family. Our findings expand the known diversity of equine papillomaviruses and contribute valuable insights into their evolutionary relationships.

**Supplementary Information:**

The online version contains supplementary material available at 10.1007/s11262-025-02190-y.

## Introduction

Members of the *Papillomaviridae* family are small non-enveloped viruses with double-stranded circular DNA genomes. This diverse viral family consists of two subfamilies: *Firstpapillomavirinae* and *Secondpapillomavirinae*. The *Firstpapillomavirinae* comprise over 50 genera and more than 130 species, while *Secondpapillomavirinae* includes only a single genus and species [1]. Papillomaviruses typically exhibit high host specificity and have been identified in a wide range of mammalian, reptile, bird and fish hosts [2]. Infections are often subclinical but can manifest as lesions, benign diseases, or even malignant cancers [3, 4]. While human papillomaviruses are the most extensively studied and genetically characterized, advances in techniques such as rolling-circle amplification combined with high-throughput sequencing (HTS) have significantly increased the detection and whole-genome characterization of animal papillomaviruses [2, 5, 6]. In equines, ten *Equus caballus* papillomaviruses (EcPV1 to 10) and two *Equus asinus* papillomaviruses (EaPV1 and EaPV2) have been identified (according to the Papillomavirus Episteme, PaVE). Interestingly, despite the generally high host specificity of papillomaviruses, bovine papillomavirus types 1, 2, and 13 have been shown to infect horses and cause equine sarcoids [7, 8].

In this paper, we describe the detection and genetic characterization of a divergent equine papillomavirus identified through a viral metagenomic investigation of an 18-year-old Frederiksborg mare admitted to the Large Animal Teaching Hospital (LATH) with anamnesis of ataxia and with neurological signs compatible with vestibular disease. The mare was hospitalized for a duration of six days and was treated symptomatically and sent home without further diagnostic than biochemistry and hematology taken at admission and again 3 days later. At admission creatinine kinase (CK, 2141 U/L) and aspartate aminotransferase (AST, 441 U/L) was found elevated, at follow up three days later, only the concentration of AST was found elevated (488 U/L). No other diagnostic test was performed, and the mare was sent home after 6 days of hospitalization and lost to follow up.

Nasal swab and serum samples were collected by authorized personnel at the LATH. Ethical approval for the procedures was obtained from the University of Copenhagen, LATH Ethical committee (permit no. 2022-009) before any samples were collected. Viral metagenomic analysis was performed using the same methodology and conditions as described in one of our previous publications [9]. Briefly, serum and nasal swab samples were filtered (0.45 µm) to remove potential bacteria. From half of each sample, RNA was extracted using a combination of TRIzol (Invitrogen) and the GeneJET RNA Purification Kit (Thermo Fisher Scientific), followed by ribosomal RNA depletion with the Ribo-Zero Plus rRNA Depletion Kit (Illumina). The other half of each sample, after filtration, was treated with RNase and DNase to reduce host nucleic acids, while viral nucleic acids remain protected within the viral capsid. DNA was then extracted using the GeneJET Genomic DNA Purification Kit (Thermo Fisher Scientific). The RNA and DNA extracted from the serum and nasal swab were then randomly amplified, and the purified products were pooled. Nanopore sequencing was conducted using the Native Barcoding Kit 24 V14 (Oxford Nanopore Technologies) to construct the sequencing library. Sequencing was performed on one flow cell (R10.4.1) for approximately 18 h with live base calling.

Approximately 5 million reads (BioProject PRJNA1251643) were obtained, with an average read length of 422 nucleotides (nt). De novo assembly was performed using CLC Genomics Workbench (v24.0) (Qiagen), resulting in 95,231 contigs, while 621,078 reads remained unassembled (singletons). The contigs and singletons were annotated using blastx (*E* value ≤ 0.0001) via Diamond (v2.0.14). Only a few viruses infecting mammals were identified, with the most prevalent mammalian virus being Equine Herpesvirus type 2 (EHV-2). The detection of EHV-2 was expected, as this virus is known to have a global distribution [10–13] including Northern Europe [9] and is commonly found in both symptomatic and asymptomatic horses [14]. Additionally, 39 sequences (15 contigs and 24 singletons) were classified as belonging to the *Papillomaviridae* family, with most showing closest similarity to various equine papillomaviruses. However, the protein similarity was relatively low, ranging from 35 to 70%, and no complete genome sequence was recovered. A papillomavirus PCR, using Platinum SuperFi PCR Master Mix (Invitrogen), following the manufacturer’s instructions, was performed on DNA extracted from the serum and nasal swab samples. The analysis showed that the papillomavirus reads/sequences originated from the nasal swab. Therefore, DNA extracted from the nasal swab was used for further analysis. Given the divergence of the detected papillomavirus compared to known equine papillomaviruses, we proceeded with whole-genome sequencing. The majority of sequences matched the E1 gene, with some also aligning to genes encoding E2, L2, and L1. Based on these sequences, primers were designed to amplify the complete viral genome using Platinum SuperFi PCR Master Mix (Invitrogen), following the manufacturer’s instructions. PCR products were purified using the GeneJET PCR Purification Kit (Thermo Fisher Scientific) and sequenced via Sanger sequencing at Macrogen Europe. Sequences were trimmed and assembled using CLC Genomics Workbench, resulting in the complete equine papillomavirus genome (PV540703). The assembled equine papillomavirus genome was 7767 nt in length and was annotated using the Papillomavirus Annotation Tool (PuMA v1.2.2) [15]. Open reading frames (ORFs) for E6, E7, E1, E2, E4, L2, and L1 were identified. Papillomavirus genomes generally range from approximately 5700–8600 nt [1]. Despite considerable genetic diversity within the family, genome organization is highly conserved. All papillomaviruses possess the core ORFs E1, E2, L2, and L1, along with an untranslated long control region (LCR) situated immediately downstream of L1. Most papillomaviruses also encode E4 and one or more of the proteins E5, E6, and E7 [2, 16]. While L2 and L1 are structural proteins, the E proteins are non-structural and are critical for replication and oncogenesis. Additionally, alternative splicing is commonly observed [17], with E4 often translated from the spliced form E1^E4 [18]. The PuMA analysis identified the alternatively spliced transcript forms E1^E4 and E8^E2 in the genome of the characterized PV. The E8^E2 protein functions as a viral repressor and plays an essential role in regulating the papillomavirus life cycle [19, 20]. The previous mentioned LCR, contains various elements involved in regulating viral replication and transcription [21]. Within this region, PuMA identified one E1 binding site and three E2 binding sites.

**Fig. 1:**
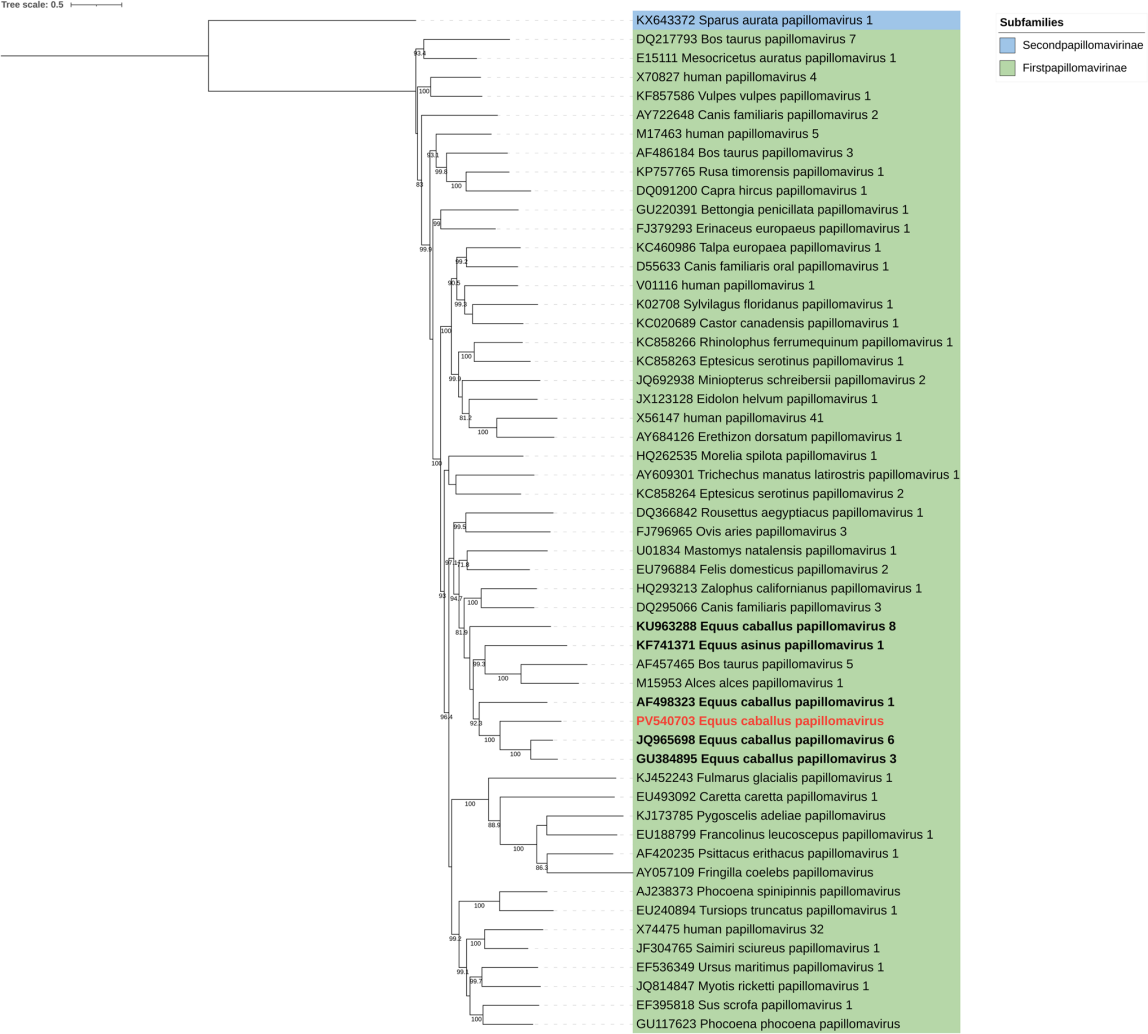
Phylogenetic analysis of *Papillomaviridae*. Maximum likelihood tree of representative member species from each genus within the *Papillomaviridae* family. The tree was constructed using concatenated E1, E2, L2, and L1 gene sequences with the Best-fit model (GTR + F + I + G4) selected based on the Bayesian Information Criterion (BIC). Bootstrap analysis was performed with 1000 ultrafast bootstrap replicates, and bootstrap values ≥ 70 are displayed. Colored labels indicate different subfamilies. The sequence obtained in this study (PV540703) is highlighted in bold red, while other papillomaviruses from *Equus* are shown in bold

Papillomavirus genera are commonly classified through phylogenetic analysis of concatenated E1, E2, L2, and L1 sequences [1]. Concatenated sequences for 53 isolates, representing one from each type genera, were downloaded from the ICTV *Papillomaviridae* resource page. The sequences, including the one obtained in this study, were aligned using MUSCLE [22] and a maximum likelihood tree was constructed using IQ-TREE (v1.6.12) [23, 24] with 1,000 ultrafast bootstrap replicates and the best substitution model determined by the lowest Bayesian Information Criterion (BIC) score. Tree visualization was performed using Interactive Tree of Life (iTOL) v7.0 [25]. The virus identified in this study clustered within the same clade as EcPV3 (genus *Dyoiotapapillomavirus*) and EcPV6 (genus *Dyorhopapillomavirus*) but was situated on a distinct separate branch (Fig. 1). In addition, phylogenetic analyses of the whole genome (Fig. S1) and of the L1 sequence (Fig. S2) from the same type species were performed, yielding similar results.

Complete genome comparison of the EcPV identified in this study revealed approximately 52% nucleotide sequence identity across the entire genome to EcPV3 and EcPV6 and 46% nucleotide sequence identity to EcPV1. In comparison, EcPV3 and EcPV6 share 66% nucleotide sequence identity to each other and EcPV1 display a 47% nucleotide sequence identity to EcPV3 and EcPV6. Analysis of individual genes demonstrated that the highest sequence identity was observed for the E1 and L1 genes (Table 1). For the L1 gene, which is commonly used for papillomavirus classification, the virus identified in this study exhibited 58.4%, 60.0% and 52.6 nucleotide sequence identity to EcPV3, EcPV6 and EcPV1, respectively. In contrast, the L1 sequence identity between EcPV3 and EcPV6 was 70.2% and their nucleotide sequence identity to EcPV1 was approximately 57%. The L1 amino acid identities were similar to the L1 nucleotide sequence identities.

**Table 1:** Sequence comparison

		Viral genes/proteins						
		E6	E7	E1	E2	E4	L2	L1
Nucleotide identity (%)	EcPV3	43.3	51.6	62.3	51.2	45.4	49.1	58.4
	EcPV6	42.7	50.2	61.4	50.1	38.7	48.7	60.0
	EcPV1	42.6	37.5	58.4	45.8	29.2	37.9	52.6
Amino acid identity (%)	EcPV3	32.0	34.7	58.4	35.4	29.3	41.8	57.1
	EcPV6	32.0	38.1	59.4	35.4	24.9	41.9	58.3
	EcPV1	30.6	25.5	50.1	34.2	14.9	29.5	47.2

The table presents the nucleotide and amino acid sequence identity (in percentage) of the virus characterized in the present study (PV540703) compared to Equus caballus papillomavirus 3 (GU384895), Equus caballus papillomavirus 6 (JQ965698) and Equus caballus papillomavirus 1 (AF498323). Sequence comparison was performed across all individual genes/proteins

In summary, we have identified and performed whole-genome characterization of a novel papillomavirus detected in a nasal swab from a horse in Denmark. The papillomavirus was identified in a nasal swab and is most likely a bystander rather than the causative agent of the observed neurological signs. No cerebrospinal fluid or brain tissue samples were available for analysis. However, the identified virus displayed high sequence diversity compared to other genetically characterized equine papillomaviruses. The combination of phylogenetic analysis (Fig. 1) and the low viral gene sequence identity observed compared to the closest genera type species (Table 1) suggests that this virus may potentially represent a novel genus or species within the *Papillomaviridae* family.

## Supplementary Information

Below is the link to the electronic supplementary material.

Supplementary file1 (PDF 112 KB)

## Data Availability

The Nanopore sequencing reads have been submitted to Sequence Read Archive (SRA; NCBI) under the BioProject PRJNA1251643 and the complete papillomavirus genome has been submitted to GenBank (accession number PV540703).
