# Supplementary material for: Identification and whole-genome characterization of a novel equine papillomavirus
Source: Virus Genes. 2025 Oct 23;61(6):747–51. doi: 10.1007/s11262-025-02190-y (PMC12678564; doi:10.1007/s11262-025-02190-y)
Supplement: Supplementary file 1 — Supplementary file1 (PDF 112 KB) [file 11262_2025_2190_MOESM1_ESM.pdf]

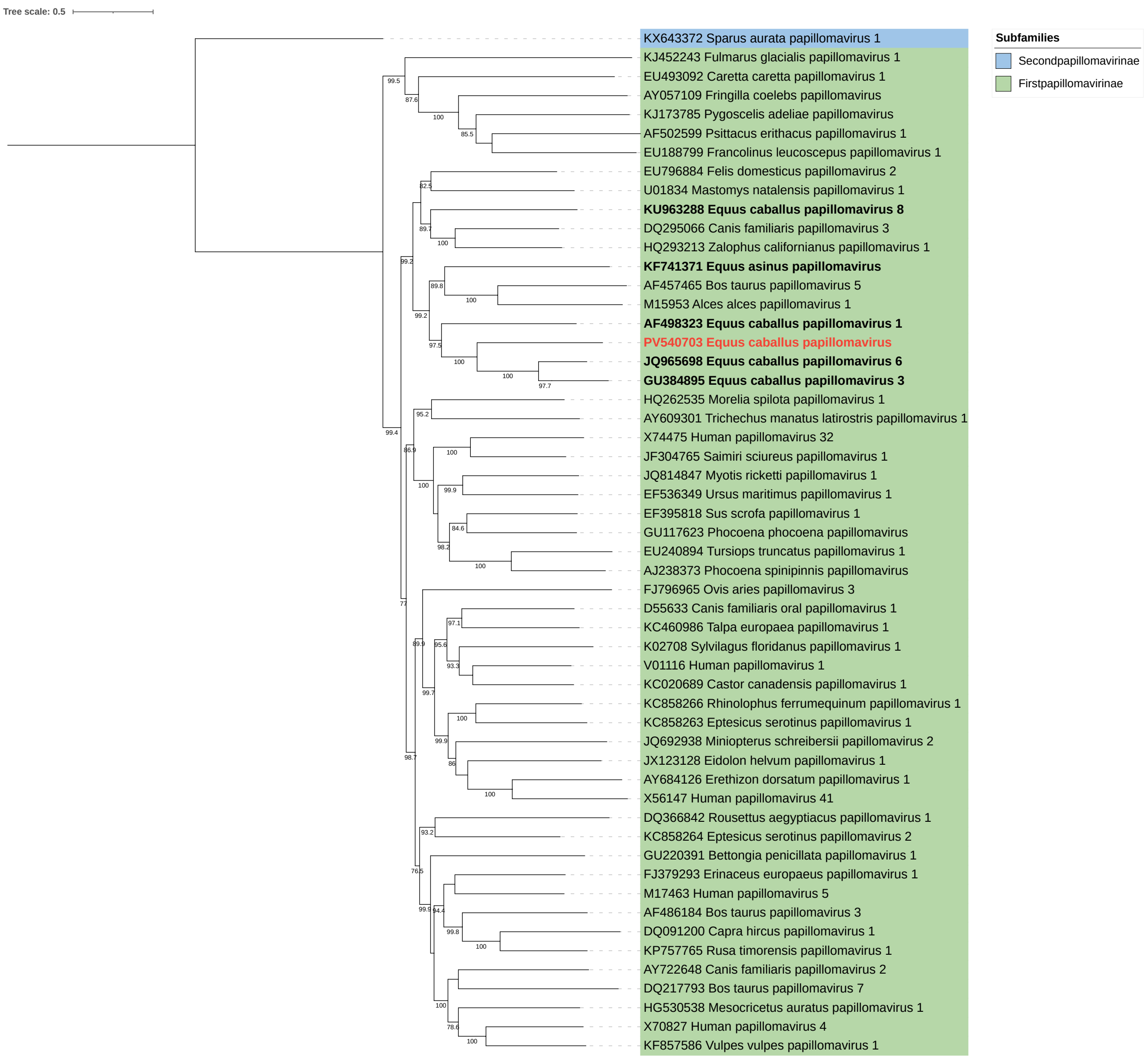

Figure S1 Whole genome phylogenetic analysis of *Papillomaviridae*. Maximum likelihood tree of representative member species from each genus within the *Papillomaviridae* family. The tree was constructed using with the Best-fit model (GTR+F+I+G4) selected based on the Bayesian Information Criterion (BIC). Bootstrap analysis was performed with 1,000 ultrafast bootstrap replicates, and bootstrap values ≥ 70 are displayed. Colored labels indicate different subfamilies. The sequence obtained in this study (PV540703) is highlighted in bold red, while other papillomaviruses from *Equus* are shown in bold.

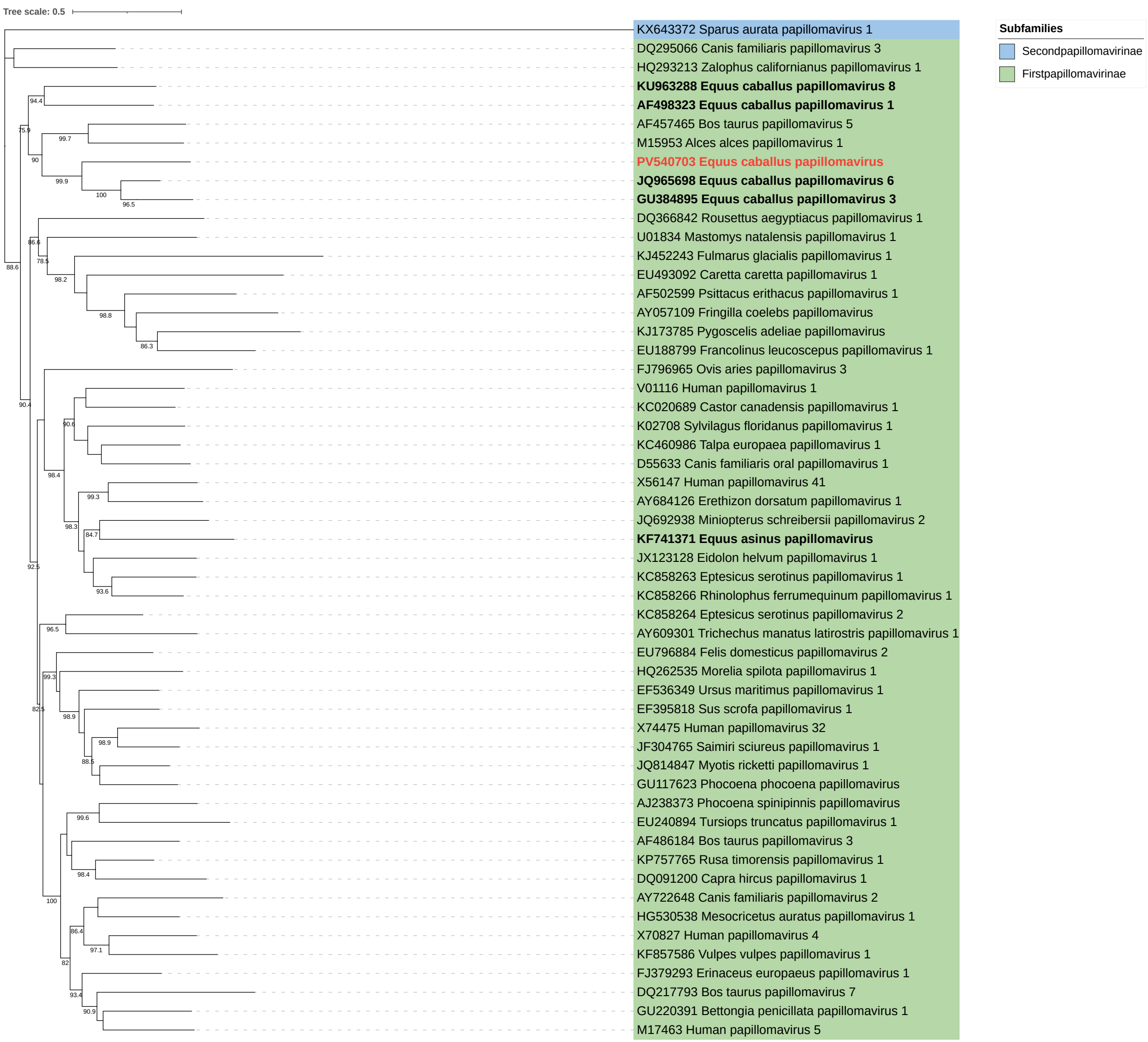

Figure S2 L1 phylogenetic analysis of *Papillomaviridae*. Maximum likelihood tree of representative member species from each genus within the *Papillomaviridae* family. The tree was constructed using the L1 gene with the Best-fit model (GTR+F+I+G4) selected based on the Bayesian Information Criterion (BIC). Bootstrap analysis was performed with 1,000 ultrafast bootstrap replicates, and bootstrap values ≥ 70 are displayed. Colored labels indicate different subfamilies. The sequence obtained in this study (PV540703) is highlighted in bold red, while other papillomaviruses from *Equus* are shown in bold.
